# Therapeutic drug monitoring (TDM) of β-lactam/β-lactamase inhibitor (BL/BLI) drug combinations: insights from a pharmacometric simulation study

**DOI:** 10.1093/jac/dkae375

**Published:** 2024-10-22

**Authors:** Amaury O’Jeanson, Elisabet I Nielsen, Lena E Friberg

**Affiliations:** Department of Pharmacy, Uppsala University, Uppsala, Sweden; Department of Pharmacy, Uppsala University, Uppsala, Sweden; Department of Pharmacy, Uppsala University, Uppsala, Sweden

## Abstract

**Background:**

The emergence of β-lactamase-producing bacteria has led to the use of β-lactam (BL) antibiotic and β-lactamase inhibitor (BLI) drug combinations. Despite therapeutic drug monitoring (TDM) being endorsed for BLs, the impact of TDM on BLIs remains unclear.

**Objectives:**

Evaluate whether BLIs are available in effective exposures at the site of infection and assess if TDM of BLIs could be of interest.

**Methods:**

Population pharmacokinetic models for 9 BL and BLI compounds were used to simulate drug concentrations at infection sites following EMA-approved dose regimens, considering plasma protein binding and tissue penetration. Predicted target site concentrations were used for probability of target attainment (PTA) analysis.

**Results:**

Using EUCAST targets, satisfactory (≥90%) PTA was observed for BLs in patients with typical renal clearance (CrCL of 80 mL/min) across various sites of infection. However, results varied for BLIs. Avibactam achieved satisfactory PTA only in plasma, with reduced PTAs in abdomen (78%), lung (73%) and prostate (23%). Similarly, tazobactam resulted in unsatisfactory PTAs in intra-abdominal infections (79%), urinary tract infections (64%) and prostatitis (34%). Imipenem-relebactam and meropenem-vaborbactam achieved overall satisfactory PTAs, except in prostatitis and high-MIC infections for the latter combination.

**Conclusions:**

This study highlights the risk of solely relying on TDM of BLs, as this can indicate acceptable exposures of the BL while the BLI concentration, and consequently the combination, can result in suboptimal performance in terms of bacterial killing. Thus, dose adjustments also based on plasma concentration measurements of BLIs, in particular for avibactam and tazobactam, can be valuable in clinical practice to obtain effective exposures at the target site.

## Introduction

The emergence of β-lactamase-producing bacteria significantly challenges the efficacy of β-lactam (BL) antibiotics, which are widely prescribed in hospital settings. To restore the activity of BL drugs against resistant pathogens, a BL compound may be used in combination with a β-lactamase inhibitor (BLI) drug. There are currently multiple approved BL/BLI combinations, and several additional combinations are in development or awaiting approval.^1^ However, achieving optimal drug exposure at the site of infection remains a critical concern, particularly given the substantial variability in patient pharmacokinetics (PK). This variability is especially pronounced in critically ill patients, where factors such as organ dysfunction and altered drug metabolism significantly affect drug levels and therapeutic outcomes.^2^

Although BL dose adjustments based on estimated renal function are often recommended, several studies have highlighted that due to high variability between patients, BL exposures are frequently suboptimal.^3–6^ As a result, there has been a growing interest in the use of therapeutic drug monitoring (TDM) for BL antibiotics, particularly in critically ill patients and those with severe infections.^7–10^ Although TDM presents many challenges in its implementation, it is increasingly recommended in clinical practice to optimize BL exposure,^11^ primarily to avoid treatment failure, and to address toxicity issues.^12^ TDM focuses currently on optimizing the BL exposure, while there is limited insight into the target attainment of the accompanying BLI drug. Clinically established pharmacodynamic (PD) targets for BLIs are currently lacking, making it challenging to ascertain whether exposure to BLIs is sufficient to inhibit β-lactamases effectively. However, the available PK/PD targets for BLIs, derived mainly from preclinical studies, have been crucial in drug development and were successfully used to support market approval for BL/BLI combinations. These preclinical targets are expected to be robust enough to guide treatment strategies in the absence of clinical targets.

If the monitored BL exposure is acceptable, can it be assumed that the exposure to the accompanying BLI is also sufficient? To comprehensively address this question, pharmacometric modelling and simulation can be utilized. This approach provides a controlled setting to explore various scenarios and factors that may be impractical or ethically challenging in a clinical trial. Probability of target attainment (PTA), quantifies the likelihood of a drug achieving a specific PD target under given dose regimens. Using population pharmacokinetic (PopPK) models for each compound extracted from the literature, concentrations at the site of infection can be predicted to derive PTA for the BL drug alone, the BLI drug alone, and for the combination as a whole. Focusing on five approved BL/BLI combinations for medical use—piperacillin-tazobactam (PIP-TAZ), ceftolozane-tazobactam (CET-TAZ), ceftazidime-avibactam (CAZ-AVI), meropenem-vaborbactam (MER-VAB) and imipenem-relebactam (IMI-REL)—this study aimed to evaluate whether BLI compounds are expected to be present in effective exposures at the site of infection and to assess if TDM of BLI drugs could be of interest in clinical practice.

## Materials and methods

### Overview

This study employed a comprehensive pharmacometric simulation framework to predict drug concentration-time profiles for nine BL antibiotics and BLIs across different clinical scenarios. Previously established PopPK models, and associated parameters (Table S1, available as Supplementary data at *JAC* Online), for each compound were implemented in the simulation software. The simulations were conducted following the dosing regimens outlined in the European Summary of Product Characteristics (SmPC) for each BL/BLI combination, corresponding to their approved therapeutic indications (Table S2).

The investigated therapeutic indications were complicated intra-abdominal infection (cIAI), hospital-acquired pneumonia or ventilator-associated pneumonia (HAP/VAP), complicated urinary tract infection (cUTI, including acute pyelonephritis), and associated bacteraemia. Additionally, prostatitis was included as an infection with a low penetration site, despite not being included in the SmPCs.

To predict unbound drug concentrations at infection sites, total plasma concentrations were adjusted for unbound fractions (fu) and tissue penetration ratios (TPR), listed in Table 1. The derived target site concentrations mimicked epithelial lining fluid (ELF) for lung infections, peritoneal fluid for cIAI, prostate concentrations for prostatitis, and unbound plasma concentrations for bacteraemia and pyelonephritis. If a TPR value was unavailable for a BLI, the value for the corresponding BL was used given their similar physicochemical and PK properties.

**Table 1.** Summary of unbound fractions and organ penetration ratios for each compound of the simulation framework, extracted from its respective SmPC and the literature

Drug	Unbound fraction in plasma	Lung penetration ratio (ELF)	Abdomen penetration ratio (peritoneal fluid)	Prostate penetration ratio
Avibactam	0.92^13^	0.30^13^	0.33 (extrapolated)	0.15 (extrapolated)
Ceftazidime	0.90^13^	0.30^13^	0.33–0.35^14^	0.15^15^
Ceftolozane	0.79–0.84^16^	0.50^16^	0.74^17^	Unknown
Imipenem	0.80^18^	0.55^18^	0.82^19^	Unknown
Meropenem	0.98^20^	0.65^20^	0.92^21^	0.15^22^
Piperacillin	0.70^23^	0.49^24^	0.75^25^	0.35–0.37^26^
Relebactam	0.78^18^	0.54^18^	0.82 (extrapolated)	Unknown
Tazobactam	0.70^16,23^	0.62^16^ & 1.21^24^	0.79^25^ & 0.95^17^	0.31–0.37^26^
Vaborbactam	0.67^20^	0.79^20^	0.92 (extrapolated)	0.15 (extrapolated)

&: For tazobactam, values are reported as separate values rather than a range since values come from different drug combinations (CET-TAZ or PIP-TAZ). For detailed analysis of the impact of swapping TPR values between CET-TAZ and PIP-TAZ on PTA, please refer to Table S4.

### Simulation framework

Suitable PopPK models for each compound were searched for in PubMed. Studies that included models for both the BL and BLI were prioritized.^27–30^ Models from separate publications were selected for the PIP-TAZ combination since no joint publication was identified.^31,32^ Interindividual variability and any reported correlation between parameters in the models were implemented in the code. Individual concentration-time profiles were simulated from the models using two renal function scenarios: a typical renal clearance (TRC) population with a creatinine clearance (CrCL) of 80 mL/min,^33^ and an augmented renal clearance (ARC) population with a CrCL of 200 mL/min.

The PTA for each simulation scenario was obtained by comparing the target site concentration-time profiles with the PK/PD targets listed in Table 2. The targets, established using minimum inhibitory concentration (MIC) for the BL/BLI combination in the presence of a fixed concentration of the BLI, were sourced from EUCAST (European Committee on Antimicrobial Susceptibility Testing).^34–38^ Two additional scenarios with increased PK/PD targets were investigated: targets for patients with severe infections and aggressive targets.

**Table 2.** Summary of PK/PD targets for each BL/BLI combination (extracted from EUCAST’s rationale documents) and MIC increments used for computation of PTA

BL/BLI drugs	PK/PD target	EUCAST	Severe	Aggressive	MIC_BL/BLI_ (mg/L)
Ceftazidime	%*fT *> MIC	50%	75%	100%	1–2–4–**8**
Avibactam	%*fT *> C_T_^a^	50%	75%	100%	
Ceftolozane	%*fT *> MIC	30%	45%	60%	0.5–1–2–**4**
Tazobactam	%*fT *> C_T_^a^	20%	30%	45%	
Imipenem	%*fT *> MIC	6.5%	15%	35%	0.25–0.5–1–**2**
Relebactam	*f*AUC_0-24h_/MIC	≥5.2	≥8	≥12	
Meropenem	%*fT *> MIC	45%	70%	100%	1–2–4–**8**
Vaborbactam	*f*AUC_0-24h_/MIC	≥35	≥50	≥65	
Piperacillin	%*fT *> MIC	40%	70%	100%	1–2–4–**8**
Tazobactam	%*fT *> C_T_^a^	40%^b^	70%	100%	

In bold: non-species related breakpoints. EUCAST: PK/PD targets sourced from EUCAST rationale documents for BL/BLI combinations; Severe: PK/PD targets for patients with a severe infection; Aggressive: aggressive PK/PD targets.

^a^Threshold concentration (C_T_) of 1 mg/L.

^b^The defined portion of the dosing interval for the TAZ target was extrapolated since unspecified in the rationale document.

### Software and tools

PopPK models originally coded in NONMEM were transposed to C++ for compatibility with the R package mrgsolve (version 1.1.1).^39^ Simulations, PTA calculations, and data visualization were conducted in R (v.4.2.1) using mrgsolve, dplyr (v.1.3.1), and ggplot2 (v.3.5.0) packages, respectively.

For detailed descriptions of the model selection process, sensitivity analyses, and additional simulation parameters, please refer to Supplementary data.

## Results

### PTA for the BL antibiotic drugs alone

**Figure 1.**
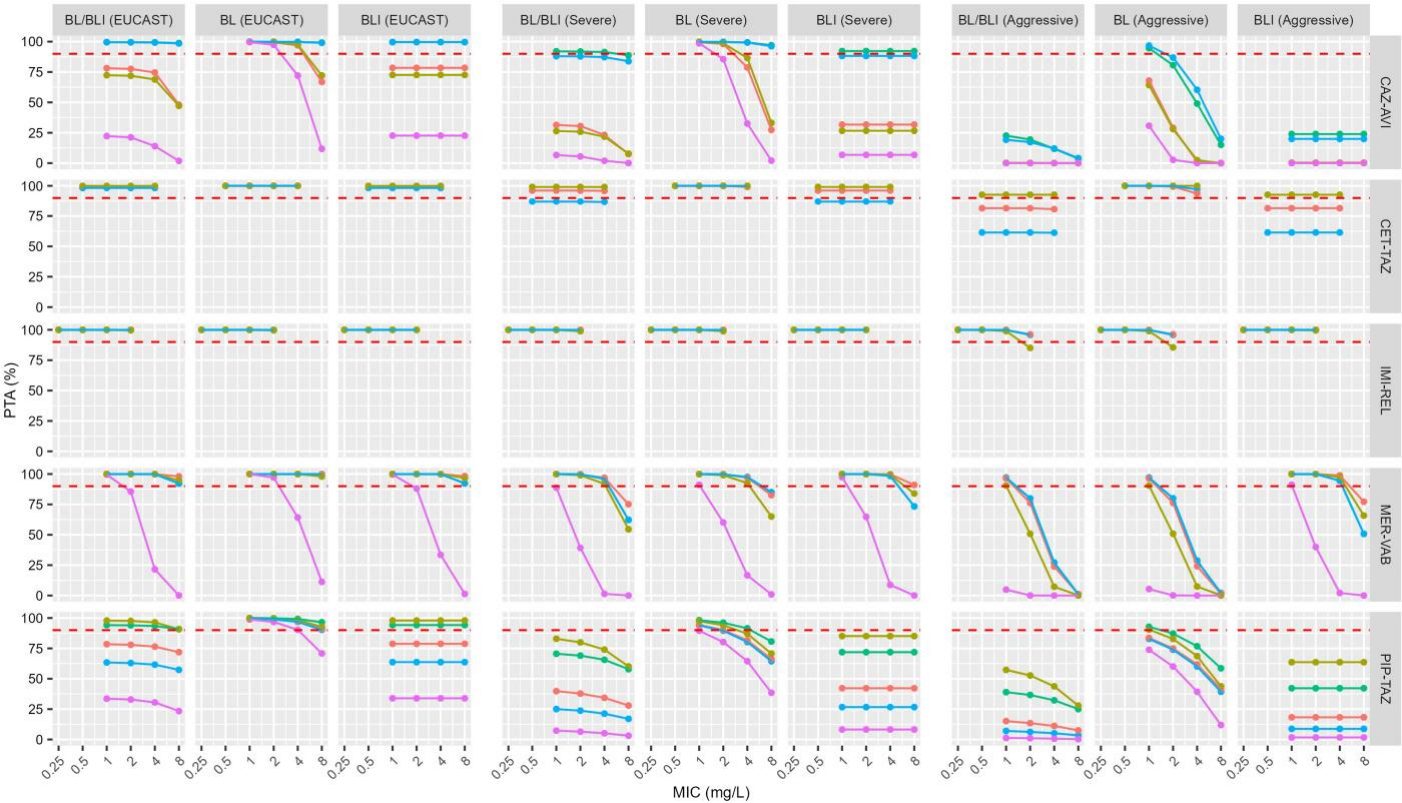
PTA results for CAZ-AVI, CET-TAZ, IMI-REL, MER-VAB and PIP-TAZ in the TRC population. PTA results are stratified by PK/PD targets (EUCAST, Severe and Aggressive) and outputted for the BL/BLI combination as a whole, the BL drug alone and the BLI drug alone. Therapeutic indications are: cIAI (orange), hospital-acquired pneumonia and ventilator associated pneumonia (mustard), cUTI (including pyelonephritis; cyan), associated bacteraemia (green), and prostatitis (purple). Red dashed line represents 90% PTA threshold. This figure appears in colour in the online version of *JAC* and in black and white in the print version of *JAC*.

Using EUCAST targets and reported values for fu and TPR in the TRC population, PTA was overall adequate (≥90%) for the recommended BL dose regimens (Figure 1) across the evaluated infection types. An exception was for prostatitis at the highest MIC_BL/BLI_ (8 mg/L) with PTA values of 12%, 11%, and 71% for ceftazidime, meropenem, and piperacillin, respectively. Furthermore, for TRC patients with lung infections or cIAI, the ceftazidime PD target alone was not reached at an MIC_CAZ/AVI_ of 8 mg/L.

As expected for renally-cleared drugs, the ARC population (Figure S1) had lower PTAs than the TRC population. PTAs for prostate infections were insufficient for MIC_BL/BLI_ ≥ 4 mg/L in the case of ceftazidime and meropenem and for MIC_PIP/TAZ_ ≥ 1 mg/L in the case of piperacillin. In the ARC population, PTA for the piperacillin target alone was only ≥90% for bacteraemia and pneumonia populations when MIC_PIP/TAZ_ was low (1 mg/L).

Also as anticipated, higher PK/PD targets resulted in lower PTA (columns 5 and 8 in Figure 1). For example, for lung infection (MIC_MER/VAB_ of 2 mg/L) in the TRC population, the recommended BL dose regimen led to ≥99% PTA with the EUCAST target (45% *fT *> MIC) and the target for severe infections (70% *fT *> MIC), but reduced to 51% with the aggressive target (100% *fT *> MIC).

### PTA for the inhibitor of β-lactamase drugs alone

The time-dependent BLIs (avibactam and tazobactam) had overall lower PTA than the AUC-based BLIs (relebactam and vaborbactam) for EUCAST targets and the dose regimens described in SmPCs. As for the BL alone, the PTAs were lower in the ARC than in the TRC population.

PTA results for time-dependent BLIs were identical across all four tested MIC_BL/BLI_ values, as targets were based on a MIC-independent threshold concentration (C_T_ of 1 mg/L). For EUCAST targets, PTA was adequate for avibactam in bacteraemia and cUTI but <90% for other infections. When combined with ceftolozane, the target suggested for tazobactam of 20% *fT *> C_T_, resulted in satisfactory PTA for both TRC and ARC populations in all infection types, but was just below the threshold for cUTI in ARC (89% PTA). The tazobactam target of 40% *fT *> C_T_ (when combined with piperacillin) was only achieved in ≥90% of the patients in the case of bacteraemia or HAP/VAP in the TRC population. In ARC patients, the PTAs of tazobactam were all <90% (67% PTA against bacteraemia, 82% PTA against HAP/VAP, 37% PTA against cIAI and 6% against prostatitis).

Relebactam dose regimens led to 100% PTA in all investigated infection types, renal clearance populations and target magnitudes. PTA results for vaborbactam were more disparate. For example, for high-MIC HAP/VAP (8 mg/L) in the TRC population, the recommended BLI dose regimen resulted in ≥90% PTA with the EUCAST target (*f*AUC/MIC ≥ 35), but <90% PTA with the severe (*f*AUC/MIC ≥ 50) and aggressive (*f*AUC/MIC ≥ 65) targets.

### PTA for the BL/BLI combinations

PTAs for BL/BLI combinations were always the same or lower than PTAs observed for BL or BLI since both targets had to be met. In most cases, it was the poor PTA achieved by BLIs that limited target achievement for the combination. For example, when using EUCAST targets in TRC patients treated with CAZ-AVI for a pneumonia infection (with a MIC_CAZ/AVI_ of 4 mg/L), PTA was 98% for ceftazidime, 73% for avibactam and 69% for the combination. For targets for severe infections, ARC patients treated with ceftozolane-tazobactam for cUTI (MIC_CET/TAZ_ of 2 mg/L) had an estimated PTA of 98% for ceftolozane, but a PTA of 61% for tazobactam and the combination. In ARC patients treated with MER-VAB for bacteraemia (MIC_MER/VAB_ of 8 mg/L), PTA was >99% for meropenem but 81% for vaborbactam and the combination, when using EUCAST targets. High PTAs (>90%) were achieved for all investigated scenarios of the IMI-REL combination.

The sensitivity analysis (Figures S2–S6) illustrated that the conclusion on reaching 90% PTA or not was only affected by the choice of fu and TPR in a few cases. For example, PTA became acceptable for cUTI (MIC of 2 mg/L) in the TRC population treated with CAZ-AVI using the severe target when the TPR was set to the high value (Figure S2).

## Discussion

A simulation framework was developed and used for PTA analysis of 5 marketed BL/BLI combinations. PTA was computed for the BL, BLI and the BL/BLI combination, based on each compound’s predicted target site concentration, PK/PD target magnitude, and patients’ renal function. Except for IMI-REL, for which overall adequate PTA was achieved, the accompanying BLI compounds (avibactam, tazobactam and vaborbactam) had target site concentrations resulting in low PTA, and consequently a low PTA for the combination. The time-dependent BLIs (avibactam and tazobactam) had in general a lower PTA, than the AUC-based BLIs (vaborbactam and relebactam). The satisfactory PTA results for IMI-REL (>99%) may be due to the relatively low PK/PD target magnitudes (6.5%–35% *fT *> MIC for imipenem and *f*AUC/MIC ≥ 5.2–12 for relebactam) and the relatively low non-specie related breakpoint of 2 mg/L. The sensitivity analysis illustrated the impact of fu and TPR on target attainment at the site of infection, demonstrating that in most cases, the impact was low and rarely altered the overall conclusions regarding whether the PD targets were achieved in at least 90% of the population or not.

The difference in PTA outcomes between time-dependent and AUC-based BLIs is partly due to the nature of their PK/PD targets. Time-dependent BLIs require maintaining the drug concentration above a threshold for a certain percentage of the dosing interval (*fT *> C_T_). The relationship to dose is non-proportional, i.e. dose adjustments do not linearly translate to improvements in PTA, complicating therapy optimization. In contrast, AUC-based BLIs are evaluated based on the *f*AUC/MIC ratio, where an increase in dose typically results in a more predictable increase in PTA.

The predicted low PTA results for the BLIs avibactam, tazobactam, and vaborbactam raise pertinent questions about the necessity of incorporating TDM for BLIs in clinical practice. While our study highlights the potential limitations in achieving effective BLI concentrations at the target site, the implications for patient outcomes warrant further exploration. TDM for antibiotics is a well-established practice. However, the relevance and feasibility of extending TDM to BLIs have not been firmly established. Considering the significant impact of the time-dependent BLIs, avibactam and tazobactam, on the overall PTA in BL/BLI combinations, suggests that TDM may indeed be of paramount interest for optimizing treatment outcomes.

Additionally, since the BL/BLI drugs are marketed as fixed-dose combinations, any adjustment in the dose of the BL would inherently change the dose of the BLI, and vice-versa. Therefore, adjusting for a suboptimal exposure of one compound might lead to excessive exposure of the other component. This underscores the importance of TDM in ensuring that both the BL and BLI achieve acceptable concentrations.

Target attainment in ARC patients treated with CAZ-AVI or PIP-TAZ was insufficient. Labels including clear dosing recommendations targeted at ARC patients would hence, based on these results, increase favourable therapeutic outcomes in these cases. With the use of population-specific PopPK models, a similar simulation approach could be extended to evaluate how renal replacement therapy or extracorporeal membrane oxygenation impacts the PTA of BL/BLI combinations and if TDM for the BLI is warranted.

This study provides a novel perspective by incorporating target site concentrations into the simulation framework, in contrast to the standard approach which typically focuses on unbound plasma concentrations.^27^ By evaluating drug exposure at the specific infection sites relevant to each therapeutic indication—such as peritoneal fluid for cIAI and ELF for pneumonia—our approach enhances the precision of target attainment predictions where bacteria reside. This method offers a more accurate assessment of drug efficacy compared with plasma-based evaluations alone.

Since clinically determined PK/PD targets for the BLIs alone and for BL-BLI combinations were not available, we applied the EUCAST targets, often considered as standards. In general, the choice of targets is frequently debated, and alternative (increased) targets are suggested based on clinical experience.^3,40,41^ For example, in the ICU, targets of 100% *fT *> MIC, or even 100% *fT *> 4×MIC, are often used for BLs in severe infections.^2,42^ Based on the PK/PD targets investigated in this work, the PTA results suggest that higher doses may be warranted (except for IMI-REL). However, further research and clinical evaluation are crucial to establish a clear relationship between drug exposure and clinical or microbiological outcomes. This is particularly important for developing consensual PK/PD targets for BLIs and BL/BLI combinations, which would be instrumental in informing TDM and model-informed precision dosing practices.

TDM for BL antibiotics is typically based on trough levels due to the generally accepted target of 100% *fT *> MIC in the ICU.^43,44^ For time-dependent BLIs, adopting a similar trough-based TDM strategy would be beneficial—limiting samples taken from patients. However, this would involve a minor adjustment in the used PK/PD targets from 20% to 50% *fT *> C_T_ to 100% *fT *> C_T_, and carry over the limits of having trough concentrations below the lower limit of quantification—uninformative for subsequent dose predictions.^45^ For AUC-based BLIs, a trough-only sampling strategy may not be adapted to estimate *f*AUC_24h_ with precision. A recent simulation study showed that precision of *f*AUC_24h_ estimates increased with a two-sampling strategy (mid-dose and trough sampling being the most favourable option).^45^

In the current study, TPR and fu values were used to predict the drug concentrations at the site of infection. The assumption of a constant TPR implies that the concentration-time profile in the tissue mirrors the shape of the PK profile observed in the originally sampled organ (typically plasma), thereby overlooking potential delays in distribution and/or drug accumulation within the tissue. The sensitivity analysis (Figures S2–S6) illustrated that the chosen parameter values affect the target site drug concentrations and, in some cases, a PTA ≥90% switched to become <90%, or vice versa. More resource-dependent methods such as physiologically-based PK models may further improve predictions of the concentration-time course in various tissues.

Except for the piperacillin model, PopPK models used in this study were built using pooled data from phase I, II and III studies. Data originating from such controlled settings may not fully reflect the broader patient population using the therapy in the real world. It is important to note that PK can vary across different patient populations, which may introduce discrepancies between the target populations used in the PopPK models and the clinical indications for our simulations. In addition, the PopPK models had been developed separately (one model for the BL and another for the BLI). Covariates were shared for an individual between the 2 models of a BL/BLI combination in the simulations, but since information on the potential correlation between random effect parameters of the 2 compounds was lacking, the PTA results may, to some degree, have been affected.^46^

Additionally, there is a need to explore alternatives to the use of plasma concentration-based PK/PD targets derived from preclinical studies to predict efficacy regardless of the target site location. Only the free drug concentrations of antibiotics at the target site are responsible for the therapeutic effect. Thus using a plasma-concentration-based approach can lead to overestimation of the target site concentrations and consequently the clinical efficacy.^47^

Further research is needed to understand the mechanistic aspects of BL/BLI combinations, considering binding kinetics, β-lactamase production and bacterial membrane permeability. An important assumption that was made in this study when assessing the PTA for the BL/BLI combinations was that both BL and BLI PK/PD targets needed to be met. For example, if the ceftazidime concentration was high enough to meet the target, but avibactam concentration was not, the target for the combination would be considered unattained. However, from a mechanistic point of view, the mechanism of action of a BL/BLI combination should be pictured as sequential (rather than simultaneous), where the BLI molecules first neutralize the produced β-lactamases leaving the producers (the bacteria) ‘defenceless’, and only then can the undegraded BL molecules exert their bactericidal activity. How far apart these 2 sequences can be for bacterial killing to actually occur has not been investigated. Avibactam, relebactam and vaborbactam have been described as having reversible binding kinetics with β-lactamases, whereas tazobactam has irreversible binding with its targets.^48–50^ Moreover, β-lactamases can either be continuously produced or their production may be induced by the presence of drugs (BL or BLI).^51^ Therefore, the β-lactamase production rate, quantity over time and variability, may impact the bacterial killing. In the absence of information, data or modelling predictions, these binding and β-lactamase turnover considerations were not accounted for in the computation of PTA for BL/BLI combinations.

The PK/PD target for time-dependent BLIs is characterized by the percentage of time the free drug concentration remains above a threshold concentration (*fT *> C_T_), with C_T_ set at 1 mg/L.^34,35,38^ In contrast, PK/PD targets for BL antibiotics depend on the percentage of time the concentration remains above the MIC_BL/BLI_ (*fT *> MIC), where the MIC_BL/BLI_ is determined using a fixed BLI concentration of 4 mg/L. This implies that a higher BLI concentration is required for achieving the BL target than for attaining the BLI target alone. Moreover, the use of a constant 4 mg/L BLI concentration in MIC determination is not reflecting actual *in vivo* conditions since BLI concentrations vary over time. This could lead to an underestimation of the BL concentration needed to achieve therapeutic efficacy. The discrepancy highlights the importance of aligned PK/PD target metrics between a BL and a BLI drug within the same combination. Therefore, careful consideration is essential to develop dosing strategies for optimizing the efficacy of BL/BLI combinations using the PK/PD target approach.

Findings from this study suggest that adhering to SmPCs for CAZ-AVI, CET-TAZ, PIP-TAZ, and to a lower extent MER-VAB, might result in ineffective BLI concentrations at the site of infection. However, further explorations into the presumed relationship between target attainment and therapeutic outcomes are needed to link the observed discrepancy in target attainment for avibactam, tazobactam and vaborbactam with unfavourable clinical and/or microbiological outcomes for patients.

### Conclusions

Our simulation framework, employing PopPK models and various PK/PD targets, highlights the risk of low PTA for BLI compounds at the target site. This, in turn, significantly impacts the overall PTA for BL/BLI combinations. Particularly, low PTAs were predicted for time-dependent BLIs like avibactam and tazobactam, which emphasizes the need for considering TDM for BLIs. While the implementation of a TDM program in the hospital poses challenges, the potential benefits of optimizing treatment outcomes merit careful consideration. Further clinical exploration is needed to understand the relationship between low BLI concentrations at the site of infection and therapeutic failure, and for the development of model-informed precision dosing strategies.

## Supplementary Material

dkae375_Supplementary_Data

## Supplementary data

Figures S1 to S6 and Tables S1 to S5 are available as Supplementary data at *JAC* Online.
