# Supplementary material for: Therapeutic drug monitoring (TDM) of β-lactam/β-lactamase inhibitor (BL/BLI) drug combinations: insights from a pharmacometric simulation study
Source: J Antimicrob Chemother. 2024 Oct 22;80(1):79–86. doi: 10.1093/jac/dkae375 (PMC11695910; doi:10.1093/jac/dkae375)
Supplement: dkae375_Supplementary_Data [file dkae375_supplementary_data.docx]

**Supplementary data**

**Article title:** Therapeutic drug monitoring (TDM) of β-lactam/β-lactamase inhibitor (BL/BLI) drug combinations: Insights from a pharmacometric simulation study

**Author names:** Amaury O’JEANSON, Elisabet I. NIELSEN, Lena E. FRIBERG

**Affiliation:** Department of Pharmacy, Uppsala University, Uppsala, Sweden

**PopPK model identification and selection process**

A comprehensive search of the PubMed database was conducted to identify population pharmacokinetic (PopPK) models describing the plasma concentration-time profiles for the following compounds: avibactam, ceftazidime, ceftolozane, imipenem, meropenem, piperacillin, relebactam, tazobactam, and vaborbactam. The identified models were implemented in a pharmacometric simulation framework.

**Search strategy and selection criteria**

- Search terms: specific combinations of keywords were used to perform the search, such as "ceftazidime AND population pharmacokinetic model”
- Inclusion criteria:
  - Publications describing PopPK models for the specified compounds
  - Publications reporting PopPK models for both compounds in combination therapy (e.g., avibactam with ceftazidime) were prioritized over those describing a model for a single compound.
  - Studies involving relevant patient populations, such as patients with infections or specific demographic groups (e.g., adults).
  - Studies with a sufficiently large sample size (e.g., typically more than 30 patients).
  - Studies with comprehensive pharmacokinetic sampling, including multiple and well-distributed time points.

**Rationale for selection**

The selection process prioritized models that were expected to provide the most comprehensive and clinically relevant data for our analysis.

- Publications that described both compounds were prioritized since they have been developed using the same methodology and group of patients.
- Studies were chosen based on the relevance of their patient populations (indication, demographics) to the indications we selected for our analyses
- A larger number of patients is generally expected to increase the reliability and generalizability of the model. Thus, larger studies were preferred.
- Studies with more comprehensive sampling schedules provide more informative data for PopPK model development and validation.

**Model Implementation**

Publications including models for both the BL and the BLI were available for all combinations except piperacillin-tazobactam.^1–4^ For piperacillin, the PopPK model developed by Udy and colleagues was selected due to its high performance in patients receiving intermittent infusion of piperacillin-tazobactam.^5,6^ For tazobactam, the model by Zhang and colleagues was used.^2^ No modifications were made to the original parameterization of models in their implementation. Additional details on the selected PopPK models and their parameterizations are summarized in Table S1.

**Dosing Regimens and Indications**

In order to mimic the approved use of the five BL/BLI combinations, the European SmPCs were followed regarding indications, modes of administration, doses and dosing intervals (see Table S2).^7–11^ The following therapeutic indications were included in the simulation framework: complicated intra-abdominal infection (cIAI), hospital-acquired pneumonia (HAP), including ventilator associated pneumonia (VAP), complicated urinary tract infection (cUTI), including acute pyelonephritis, and bacteraemia that occurs in association with any of the other infections. Prostatitis in male patients were also added despite not being included in SmPCs. The label of ceftolozane-tazobactam excludes ‘associated bacteraemia’, precluding exploration of this scenario.^8^ Similarly, imipenem-relebactam is indicated for HAP (including VAP), associated bacteraemia and infections due to aerobic Gram-negative bacteria (with limited treatment options), limiting investigations to pneumonia, bacteraemia and cIAI.^9^

**Target site predictions**

Each target site was assigned a proxy: epithelial lining fluid (ELF) concentration for lung infections (pneumonia), peritoneal fluid concentrations for cIAI, prostate concentrations for prostatitis, and unbound plasma concentrations for bacteraemia and pyelonephritis. The latter was based on the assumptions that kidneys are usually very well perfused and that all drugs included in the framework are majorly renally excreted. Target site concentrations were derived by applying tissue penetration ratio (TPR) or unbound fraction (fu) as factors to each individual’s model-predicted total plasma concentrations. Unbound plasma concentrations were obtained by applying the fu, while tissue concentrations were calculated using the TPR applied to total plasma concentrations, with direct adoption of referenced values from SmPCs. If not reported in SmPC, TPR were searched for in PubMed. The selection criteria included the type of patient population, the number of patients, and the number and timing of PK samples. If not found for the BLI compound, the TPR for the BLI and the specific organ was assumed to be the same as for the BL, given their similar physicochemical and PK properties. TPR and fu, summarized in Table 1, were implemented without between-subject variability. A sensitivity analysis in the TRC population, using fu and TPR reduced or increased by 25% compared to the reported values, was conducted to assess the impact on the PTA.

**Simulated populations**

Simulated patient populations varied in three aspects: (i) the infection type (n=3-5), (ii) the BL/BLI combination received (n=5), and (iii) the renal function (n=2). The two different renal clearance populations were: a typical renal clearance (TRC) population for which creatinine clearance (CrCL) was set at 80 mL/min, typical for critically ill patients,^12^ and an augmented renal clearance (ARC) population with a CrCL value of 200 mL/min. All patients were assumed to have a weight of 70 kg, an age of 65 year, and a height of 170 cm. For each patient population, 10,000 patients were simulated from the PopPK models using the reported typical and interindividual variability parameters.

**Pharmacodynamic targets**

The PTA for each simulation scenario was obtained by comparing the target site concentration-time profiles with the PK/PD targets in Table 2. The targets, established using minimum inhibitory concentration (MIC) for the BL/BLI combination determined with a fixed concentration of BLI, were sourced from EUCAST (European Committee on Antimicrobial Susceptibility Testing).^13–17^ Two additional scenarios with increased PK/PD targets were investigated: targets for patients with a severe infection, and aggressive targets. For BL antibiotics, the PK/PD target was the free drug concentration (or concentration at the site of infection) remaining above the MIC_BL/BLI_ for a defined portion of the dosing interval (*fT*>MIC). For avibactam and tazobactam, the target was *fT*>C_T_, with C_T_ being a threshold concentration of 1 mg/L. For relebactam and vaborbactam, the target was the ratio of the area under the unbound concentration curve over 24h and the MIC_BL/BLI_ (*f*AUC/MIC). The PTA was evaluated for four different values of MIC_BL/BLI_ for each combination – the highest MIC_BL/BLI_ being the non-species-related breakpoint as reported by EUCAST. The target was defined to be reached (i) for the BL when its *fT*>MIC_BL/BLI_ was achieved, (ii) for the BLI when either the *fT*>C_T_ or *f*AUC/MIC_BL/BLI_ target was met, and (iii) for the BL/BLI combination when both the BL and BLI targets were met.

**Table S1.** General details of selected BL and BLI PopPK models.

| Drug | Modelling data | Subjects | Model structure | Covariates |
| --- | --- | --- | --- | --- |
| Ceftazidime (CAZ)^1^ | Pooled from phase I, II and III of CAZ-AVI drug development program, with a total of 9,155 observations from 1,975 adult subjects. | Healthy volunteers (n=86); cUTI patients (n=696); HAP/VAP patients (n=412); cIAI patients (n=781). | 2-compartment model with interindividual variability (IIV) on all parameters (CL, V_C_, Q and V_P_). | Creatinine clearance (CrCL) = key covariate to predicting CL of CAZ (relationship was close to proportional); no other covariate effects on CL in phase-III patients were clinically relevant (predefined threshold ± 20%). Small covariate effects on CL were retained in the final model: indication (16% higher CL for cIAI patients compared to healthy volunteers and patients with cUTI) and racial/regional origin (Chinese patients had 9% lower CL, and non-Chinese, non-Japanese Asians had 16% lower CL than non-Asians). Covariate effects on CAZ V_C_ were: indication, Asian race (clinically relevant: 27% lower V_C_ compared to non-Asians), body weight, pyelonephritis and VAP (clinically relevant: 29.7% higher V_C_ than non-HAP patients). |
| Avibactam (AVI)^1^ | Pooled from phase I, II and III of CAZ-AVI drug development program, with a total of 13,735 observations from 2,249 adult subjects. | Healthy volunteers or subjects with renal impairment from phase I (n=345); cUTI patients (n=705); HAP/VAP patients (n=413); cIAI patients (n=786). | 2-compartment model with IIV on all parameters (CL, V_C_, Q and V_P_). | CrCL = key covariate to predicting clearance of AVI (partial power function relationship); apart from CrCL, another covariate was clinically relevant: phase III patients with an APACHE-II score > 10 (CL was 19.7% lower). Small covariate effects on CL were retained in the final model: racial/regional origin (8.65% lower CL for non-Chinese and non-Japanese Asians compared to other patients). Covariate effects on AVI V_C_ were: body weight, indication (V_C_ was 32.9% higher for patients with cIAI and 43.4% higher for patients with cUTI) and VAP status. |
| Ceftolozane (CET)^2^ | Pooled from phase I, II and III of CET-TAZ drug development program, with a total of 8,330 observations from 968 adult subjects. | Healthy volunteers or subjects with renal impairment from phase I; cUTI patients (n=242); HAP/VAP patients (n=726); cIAI patients (n=222). | 2-compartment model with first-order elimination and IIV on 3 parameters (CL, V_C_ and V_P_). | CrCL = significant covariate to predicting clearance of CET (power function relationship); end-stage renal disease (ESRD) was also clinically relevant (CL was reduced by 68% in patient with ESRD, in addition to decrease associated with reduced CrCL). ESRD was also associated with 30% higher CET V_C_ compared with patients without ESRD. Effect of body weight was significant on both V_C_ and V_P_ (allometric function relationship). Types of infection were found to be significant covariates on V_C_ (pneumonia was associated with 100% increase compared to healthy volunteers). |
| Tazobactam (TAZ)^2^ | Pooled from phase I, II and III of CET-TAZ drug development program, with a total of 5,679 observations from 835 adult subjects. | Healthy volunteers or subjects with renal impairment from phase I; cUTI patients (n=242); HAP/VAP patients (n=726); cIAI patients (n=222). | 2-compartment model with first-order elimination and IIV on 3 parameters (CL, V_C_ and V_P_). | CrCL = significant covariate to predicting clearance of TAZ (power function relationship); end-stage renal disease (ESRD) was also clinically relevant (CL was reduced by 37.4% in patient with ESRD, in addition to decrease associated with reduced CrCL). ESRD was also associated with 25.1% lower TAZ V_C_ compared with patients without ESRD. Effect of body weight was significant on both V_C_ and V_P_ (allometric function relationship). Pneumonia was found to be a significant covariate on TAZ V_C_ (117% higher than in healthy volunteers) whereas cUTI and cIAI were significant on V_P_ (25% and 34% higher, respectively). |
| Imipenem (IMI)^3^ | Pooled from phase I, II and III of IMI-REL drug development program, with a total of 4,454 observations from 815 adult subjects. | Non-infected healthy volunteers (n=231); infected patients (n=624). | 2-compartment model with first-order elimination and IIV on 3 parameters (CL, V_C_ and V_P_). | Most influential covariate for IMI CL was CrCL. Other identified covariate-parameter relationships identified for IMI were health status and body weight on V_C_ and body weight on CL. |
| Relebactam (REL)^3^ | Pooled from phase I, II and III of IMI-REL drug development program, with a total of 4,814 observations from 649 adult subjects. | Non-infected healthy volunteers (n=231); infected patients (n=624). | 2-compartment model with first-order elimination and IIV on 3 parameters (CL, V_C_ and V_P_). | CrCL was identified as a significant covariate of REL CL (highest apparent correlation of all covariate-parameter relationships). Additionally, weight was identified as a significant covariate on REL V_C_. |
| Meropenem (MER)^4^ | Pooled from phase I and III of MER-VAB drug development program, with a total of 4,264 observations from 413 adult subjects. | Non-infected healthy volunteers (n=91); infected patients (n=322). | 2-compartment model with first-order elimination and IIV on 3 parameters (CL, V_C_ and V_P_). | Model was allometry-scaled with body weight (exponent of 0.75 for CL and Q; exponent of 1.0 for V_C_ and V_P_). Estimated glomerular filtration rate (eGFR) was a significant covariate to predicting renal CL of MER (sigmoid Hill-type function relationship); age was also found to have a significant effect on renal CL. |
| Vaborbactam (VAB)^4^ | Pooled from phase I and III of MER-VAB drug development program, with a total of 4,082 observations from 414 adult subjects. | Non-infected healthy volunteers (n=93); infected patients (n=321). | 2-compartment model with first-order elimination and IIV on 3 parameters (CL, V_C_ and V_P_). | Model was allometry-scaled with body weight (exponent of 0.75 for CL and Q; exponent of 1.0 for V_C_ and V_P_). Estimated glomerular filtration rate (eGFR) was a significant covariate to predicting renal CL of VAB (sigmoid Hill-type function relationship); height and study phase were also significant covariates to CL. Body surface area had a significant impact on VAB V_C_ and study phase was significant on both V_C_ and V_P_. |
| Piperacillin (PIP)^5^ | Data from 48 sepsis patients treated with PIP-TAZ. | Sepsis patients; half were receiving PIP-TAZ for treatment of nosocomial pneumonia. | 2-compartment linear model and IIV on all parameters (CL, V_C_, Q and V_P_). | Only included covariate-parameter relationship was CrCL on PIP CL. |

**Table S2.** Indications and dosing details of simulated scenarios.

| BL/BLI combination | Therapeutic indication | Dose regimen | Labelled indication (SmPC) |
| --- | --- | --- | --- |
| CAZ-AVI^7^ | Assoc. bacteraemia | 2000/500 mg q8h as 2h-inf. | Yes |
|  | cUTI |  |  |
|  | cIAI |  |  |
|  | HAP |  |  |
|  | Prostatitis |  | No |
| CET-TAZ^8^ | cUTI | 1000/500 mg q8h as 1h-inf. | Yes |
|  | cIAI |  |  |
|  | HAP | 2000/1000 mg q8h as 1h-inf. |  |
| IMI-REL^9^ | Assoc. bacteraemia | If CrCL ≥ 90 mL/min: 500/250 mg q6h as 0.5h-inf.  If CrCL < 90 mL/min: 400/200 mg q6h as 0.5h-inf. | Yes |
|  | cUTI |  |  |
|  | cIAI |  |  |
|  | HAP |  |  |
| MER-VAB^10^ | Assoc. bacteraemia | 2000/2000 mg q8h as 3h-inf. | Yes |
|  | cUTI |  |  |
|  | cIAI |  |  |
|  | HAP |  |  |
|  | Prostatitis |  | No |
| PIP-TAZ^11^ | Assoc. bacteraemia | 4000/500 mg q6h as 0.5h-inf. | Yes |
|  | cUTI | 4000/500 mg q8h as 0.5h-inf. |  |
|  | cIAI | 4000/500 mg q8h as 0.5h-inf. |  |
|  | HAP | 4000/500 mg q6h as 0.5h-inf. |  |
|  | Prostatitis | 4000/500 mg q8h as 0.5h-inf. | No |

*cUTI: complicated urinary tract infection; cIAI: complicated intra-abdominal infection; HAP: hospital-acquired pneumonia; VAP: ventilator-associated pneumonia

**Table S3.** General details of tissue penetration ratio studies.

| Drug(s) | Tissue | Population | Dose regimen | Sampling design |
| --- | --- | --- | --- | --- |
| CAZ-AVI^7^ | Epithelial lining fluid (ELF) | Healthy subjects (n=43) | CAZ/AVI 2000/500 mg q8h as 2h-inf. (n=22) or 3000/1000 mg q8h as 2h-inf. (n=21) | Bronchoalveolar lavage (BAL) was performed once per subject, 2, 4, 6, or 8h after the last infusion |
| CAZ^18^ | Peritoneal fluid | Surgical intensive care patients with severe IAI (n=18) | 1 g IV loading dose + 4.5 g/24h continuous infusion (n=12) or 1g IV loading dose + 1.5g IV intermittent bolus (n=6) | Peritoneal exudate drawn from drainage catheters. Continuous therapy: twice daily at intervals ranging from 8 to 12h. Intermittent therapy: at 1h and 8h after the start of the infusion |
| CAZ^19^ | Prostatic gland tissue | Patients with benign prostatic hypertrophy scheduled for transurethral prostatectomy (n=24) | 2 g unique dose | Prostatic tissue sampling was performed once per subject between 30-90, 90-180, 180-300, or 300-420 min post dose |
| CET-TAZ^8^ | ELF | Healthy subjects (n=25) and critically ill patients with confirmed or suspected pneumonia (n=36) | CET/TAZ 1000/500 mg q8h as 1h-inf. | BAL was performed once per subject at 1, 2, 4, 6 or 8h after the start of infusion |
| CET-TAZ^20^ | Peritoneal fluid | Patients scheduled for elective lower gastrointestinal surgery(n=9) | CET/TAZ 1000/500mg as 1h-inf. unique dose | Sample collection was performed at the end of infusion, and at 0.5h, and every hour afterwards |
| IMI-REL^9^ | ELF | Healthy subjects (n=16) | IMI/REL 500/250 mg q6h as 1h-inf. | BAL was performed once per subject at 0.5, 1, 1.5, or 3h after the last dose |
| IMI^21^ | Peritoneal fluid | Patients scheduled for laparotomy surgery (n=10) | 500 mg q8h as 0.5h-inf | Sample collection was performed at the end of infusion, and every hour for 6h afterwards |
| MER-VAB^10^ | ELF | Healthy subjects (n=26) | MER/VAB 2/2 g q8h | BAL was performed once per subject at 1.5, 3.25, 4, 6, or 8h after the last dose |
| MER^22^ | Peritoneal fluid | Patients with inflammatory bowel diseases (n=8) | 500 mg as 0.5h-inf. unique dose | Sample collection was performed at the end of infusion, and at 1, 2, 3, 4, 5 and 6h afterwards |
| MER^23^ | Prostatic gland tissue | Patients with prostatic hypertrophy scheduled for transurethral prostatectomy (n=49) | 500 mg or 250 mg as 0.5h-inf. unique dose | Prostate tissue sampling was performed during surgery at multiple time points including 0.5 (completion of infusion), 1 and 1.5 h after initiation of MER infusion. |
| PIP-TAZ^11^ | ELF | Intubated critically ill patients with suspected or confirmed pneumonia (n=17) | PIP/TAZ 4000/500 mg q8h as 0.5h-inf. (n=13) or 4000/500 mg q12h as 0.5h-inf. (n=4) | Two intrapulmonary samples were collected from each patient. Samples were collected at 0.75 and 2h, or at 0.75 and 3.5h after initiation of the infusion. |
| PIP-TAZ^24^ | Peritoneal fluid | Patients with inflammatory bowel diseases (n=10) | PIP/TAZ 4000/500 mg as 0.5h-inf. unique dose | Sample collection was performed at the end of infusion (0.5h) and every hour until end of surgery afterwards |
| PIP-TAZ^25^ | Prostatic gland tissue | Patients with prostatic hypertrophy scheduled for transurethral prostatectomy (n=47) | PIP/TAZ 4000/500 mg as 0.5h-inf. or 2000/250 mg as 0.5h-inf. unique dose | Prostate tissue sampling was performed during surgery at multiple time points including 0.5 (completion of infusion), 1 and 1.5 h after initiation of PIP-TAZ infusion. |

**Table S4.** Impact of swapping tissue penetration ratios (TPR) of tazobactam in ceftolozane-tazobactam and piperacillin-tazobactam on PTA.

Results for the TRC population, using EUCAST targets:

| Drugs | Indication | MIC (mg/L) | TAZ TPR | PTA for BL | PTA for BLI | PTA for BL/BLI |
| --- | --- | --- | --- | --- | --- | --- |
| CET-TAZ | HAP/VAP | 0.5 | 0.62 | 100% | 99.92% | 99.92% |
|  |  | 1 |  | 100% | 99.92% | 99.92% |
|  |  | 2 |  | 100% | 99.92% | 99.92% |
|  |  | 4 |  | 100% | 99.92% | 99.92% |
|  |  | 0.5 | 1.21 | 100% | 99.99% | 99.99% |
|  |  | 1 |  | 100% | 99.99% | 99.99% |
|  |  | 2 |  | 100% | 99.99% | 99.99% |
|  |  | 4 |  | 100% | 99.99% | 99.99% |
|  | cIAI | 0.5 | 0.95 | 100% | 99.76% | 99.76% |
|  |  | 1 |  | 100% | 99.76% | 99.76% |
|  |  | 2 |  | 100% | 99.76% | 99.76% |
|  |  | 4 |  | 99.99% | 99.76% | 99.75% |
|  |  | 0.5 | 0.79 | 100% | 99.65% | 99.65% |
|  |  | 1 |  | 100% | 99.65% | 99.65% |
|  |  | 2 |  | 100% | 99.65% | 99.65% |
|  |  | 4 |  | 99.99% | 99.65% | 99.65% |
| PIP-TAZ | HAP/VAP | 1 | 0.62 | 99.91% | 92.99% | 92.90% |
|  |  | 2 |  | 99.64% | 92.99% | 92.65% |
|  |  | 4 |  | 98.42% | 92.99% | 91.48% |
|  |  | 8 |  | 92.62% | 92.99% | **86.08%** |
|  |  | 1 | 1.21 | 99.91% | 97.97% | 97.88% |
|  |  | 2 |  | 99.64% | 97.97% | 97.62% |
|  |  | 4 |  | 98.42% | 97.97% | 96.41% |
|  |  | 8 |  | 92.62% | 97.97% | 90.75% |
|  | cIAI | 1 | 0.95 | 99.63% | 95.30% | 94.95% |
|  |  | 2 |  | 98.93% | 95.30% | 94.26% |
|  |  | 4 |  | 97.06% | 95.30% | 92.53% |
|  |  | 8 |  | 91.20% | 95.30% | **86.95%** |
|  |  | 1 | 0.79 | 99.63% | **78.77%** | **78.47%** |
|  |  | 2 |  | 98.93% | **78.77%** | **77.92%** |
|  |  | 4 |  | 97.06% | **78.77%** | **76.43%** |
|  |  | 8 |  | 91.20% | **78.77%** | **71.89%** |

Results for the TRC population, using severe targets:

| Drugs | Indication | MIC (mg/L) | TAZ TPR | PTA for BL | PTA for BLI | PTA for BL/BLI |
| --- | --- | --- | --- | --- | --- | --- |
| CET-TAZ | HAP/VAP | 0.5 | 0.62 | 100% | 99.08% | 99.08% |
|  |  | 1 |  | 100% | 99.08% | 99.08% |
|  |  | 2 |  | 100% | 99.08% | 99.07% |
|  |  | 4 |  | 100% | 99.08% | 99.03% |
|  |  | 0.5 | 1.21 | 100% | 99.77% | 99.77% |
|  |  | 1 |  | 100% | 99.77% | 99.77% |
|  |  | 2 |  | 100% | 99.77% | 99.77% |
|  |  | 4 |  | 100% | 99.77% | 99.77% |
|  | cIAI | 0.5 | 0.95 | 100% | 96.11% | 96.11% |
|  |  | 1 |  | 100% | 96.11% | 96.11% |
|  |  | 2 |  | 99.98% | 96.11% | 96.10% |
|  |  | 4 |  | 99.10% | 96.11% | 95.86% |
|  |  | 0.5 | 0.79 | 100% | 94.82% | 94.82% |
|  |  | 1 |  | 100% | 94.82% | 94.82% |
|  |  | 2 |  | 99.99% | 94.82% | 94.82% |
|  |  | 4 |  | 99.75% | 94.82% | 94.67% |
| PIP-TAZ | HAP/VAP | 1 | 0.62 | 97.39% | **67.57%** | **65.83%** |
|  |  | 2 |  | 94.03% | **67.57%** | **63.44%** |
|  |  | 4 |  | **87.04%** | **67.57%** | **58.65%** |
|  |  | 8 |  | **70.71%** | **67.57%** | **47.82%** |
|  |  | 1 | 1.21 | 97.39% | **85.12%** | **82.90%** |
|  |  | 2 |  | 94.03% | **85.12%** | **79.94%** |
|  |  | 4 |  | **87.04%** | **85.12%** | **73.97%** |
|  |  | 8 |  | **70.71%** | **85.12%** | **60.16%** |
|  | cIAI | 1 | 0.95 | 94.27% | **74.50%** | **70.22%** |
|  |  | 2 |  | 90.05% | **74.50%** | **66.90%** |
|  |  | 4 |  | **81.57%** | **74.50%** | **60.83%** |
|  |  | 8 |  | **66.25%** | **74.50%** | **49.41%** |
|  |  | 1 | 0.79 | 94.27% | **42.19%** | **39.78%** |
|  |  | 2 |  | 90.05% | **42.19%** | **37.81%** |
|  |  | 4 |  | **81.57%** | **42.19%** | **34.31%** |
|  |  | 8 |  | **66.25%** | **42.19%** | **27.96%** |

Results for the TRC population, using aggressive targets:

| Drugs | Indication | MIC (mg/L) | TAZ TPR | PTA for BL | PTA for BLI | PTA for BL/BLI |
| --- | --- | --- | --- | --- | --- | --- |
| CET-TAZ | HAP/VAP | 0.5 | 0.62 | 100% | 92.68% | 92.68% |
|  |  | 1 |  | 100% | 92.68% | 92.68% |
|  |  | 2 |  | 100% | 92.68% | 92.68% |
|  |  | 4 |  | 99.98% | 92.68% | 92.66% |
|  |  | 0.5 | 1.21 | 100% | 97.47% | 97.47% |
|  |  | 1 |  | 100% | 97.47% | 97.47% |
|  |  | 2 |  | 100% | 97.47% | 97.47% |
|  |  | 4 |  | 99.98% | 97.47% | 97.46% |
|  | cIAI | 0.5 | 0.95 | 100% | **81.40%** | **81.40%** |
|  |  | 1 |  | 99.98% | **81.40%** | **81.40%** |
|  |  | 2 |  | 99.43% | **81.40%** | **81.39%** |
|  |  | 4 |  | 93.62% | **81.40%** | **80.61%** |
|  |  | 0.5 | 0.79 | 100% | **77.08%** | **77.08%** |
|  |  | 1 |  | 99.98% | **77.08%** | **77.08%** |
|  |  | 2 |  | 99.43% | **77.08%** | **77.07%** |
|  |  | 4 |  | 93.62% | **77.08%** | **76.34%** |
| PIP-TAZ | HAP/VAP | 1 | 0.62 | 90.48% | **36.69%** | **33.01%** |
|  |  | 2 |  | **82.76%** | **36.69%** | **30.34%** |
|  |  | 4 |  | **68.62%** | **36.69%** | **25.11%** |
|  |  | 8 |  | **43.79%** | **36.69%** | **16.05%** |
|  |  | 1 | 1.21 | 90.48% | **63.61%** | **57.31%** |
|  |  | 2 |  | **82.76%** | **63.61%** | **52.71%** |
|  |  | 4 |  | **68.62%** | **63.61%** | **43.73%** |
|  |  | 8 |  | **43.79%** | **63.61%** | **27.88%** |
|  | cIAI | 1 | 0.95 | **83.62%** | **44.48%** | **37.21%** |
|  |  | 2 |  | **74.91%** | **44.48%** | **33.37%** |
|  |  | 4 |  | **61.81%** | **44.48%** | **27.64%** |
|  |  | 8 |  | **41.62%** | **44.48%** | **18.64%** |
|  |  | 1 | 0.79 | **83.62%** | **18.27%** | **15.15%** |
|  |  | 2 |  | **74.91%** | **18.27%** | **13.45%** |
|  |  | 4 |  | **61.81%** | **18.27%** | **11.18%** |
|  |  | 8 |  | **41.62%** | **18.27%** | **7.53%** |

**Table S5.** Clinical breakpoint for *Pseudomonas aeruginosa* and *Enterobacterales*, and non-species related breakpoints for the 5 BL/BLI combinations – source: EUCAST.

| Drugs | *P. aeruginosa* | *Enterobacterales* | Non-species |
| --- | --- | --- | --- |
| CAZ-AVI | S ≤ 8 ; R >8 | S ≤ 8 ; R >8 | 8 |
| CET-TAZ | S ≤ 4 ; R >4 | S ≤ 2 ; R >2 | 4 |
| IMI-REL | S ≤ 2 ; R >2 | S ≤ 2 ; R >2 | 2 |
| MER-VAB | S ≤ 8 ; R >8 | S ≤ 8 ; R >8 | 8 |
| PIP-TAZ | S ≤ 0.001 ; R >16 | S ≤ 8 ; R >8 | 8 |

*All values are reported in mg/L. S: susceptible; R: resistant.

**
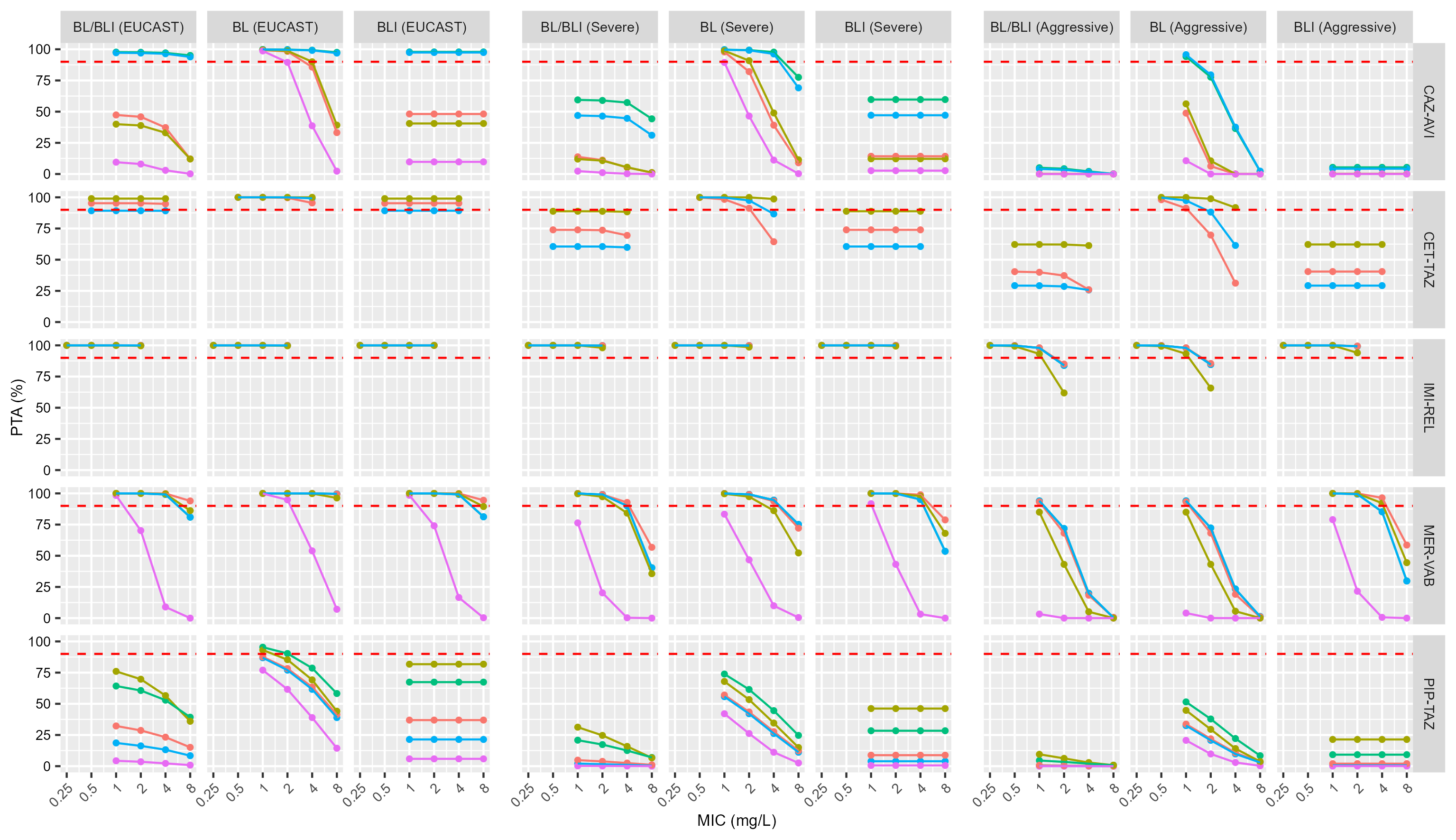
**

**Figure S1.** Probability of target attainment results for ceftazidime-avibactam (CAZ-AVI), ceftolozane-tazobactam (CET-TAZ), imipenem-relebactam (IMI-REL), meropenem-vaborbactam (MER-VAB) and piperacillin-tazobactam (PIP-TAZ) in the ARC population. PTA results are stratified by PK/PD targets (EUCAST, Severe and Aggressive) and outputted for the BL/BLI combination as a whole, the BL drug alone and the BLI drug alone. Therapeutic indications are: complicated intra-abdominal infection (orange), hospital-acquired pneumonia and ventilator associated pneumonia (mustard), complicated urinary tract infection (including pyelonephritis; cyan), associated bacteraemia (green), and prostatitis (purple). Red dashed line represents 90% PTA threshold.

**
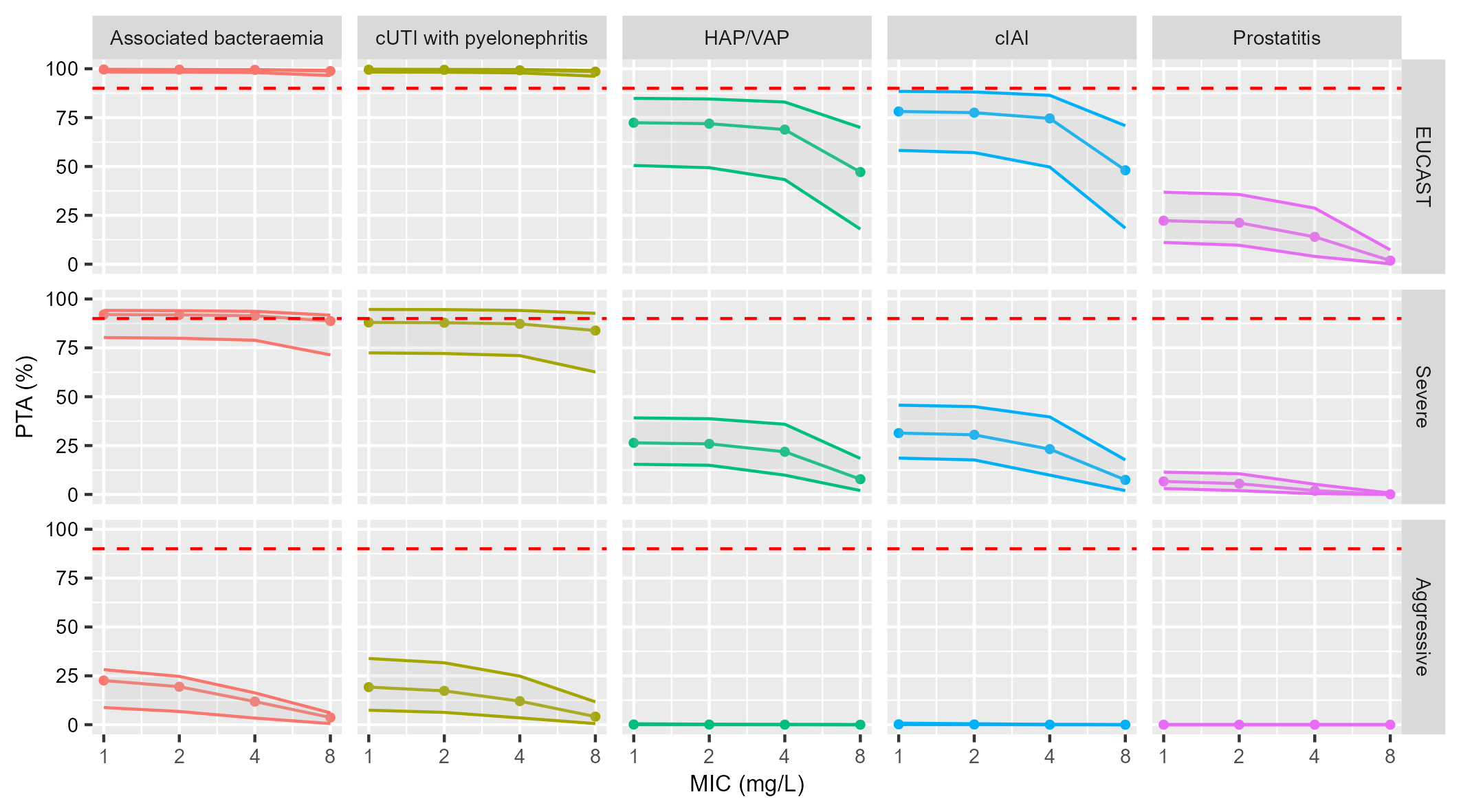
**

**Figures S2.** Probability of target attainment results for ceftazidime-avibactam (CAZ-AVI) in the TRC population, using high and low values of reported tissue penetration ratio and unbound fraction. Dotted lines correspond to reported values in the literature.

**
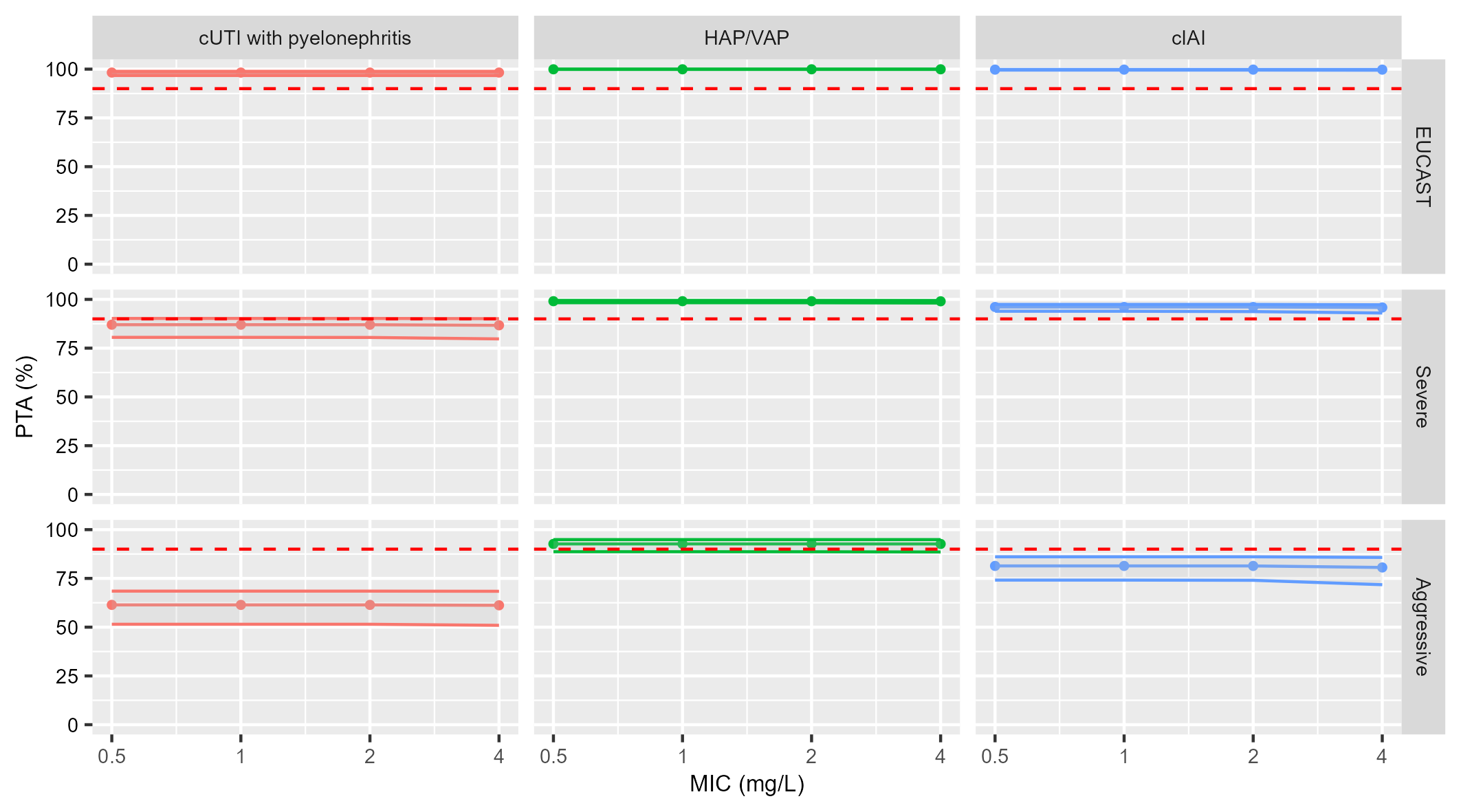
**

**Figures S3.** Probability of target attainment results for ceftolozane-tazobactam (CET-TAZ) in the TRC population, using high and low values of reported tissue penetration ratio and unbound fraction. Dotted lines correspond to reported values in the literature.

**
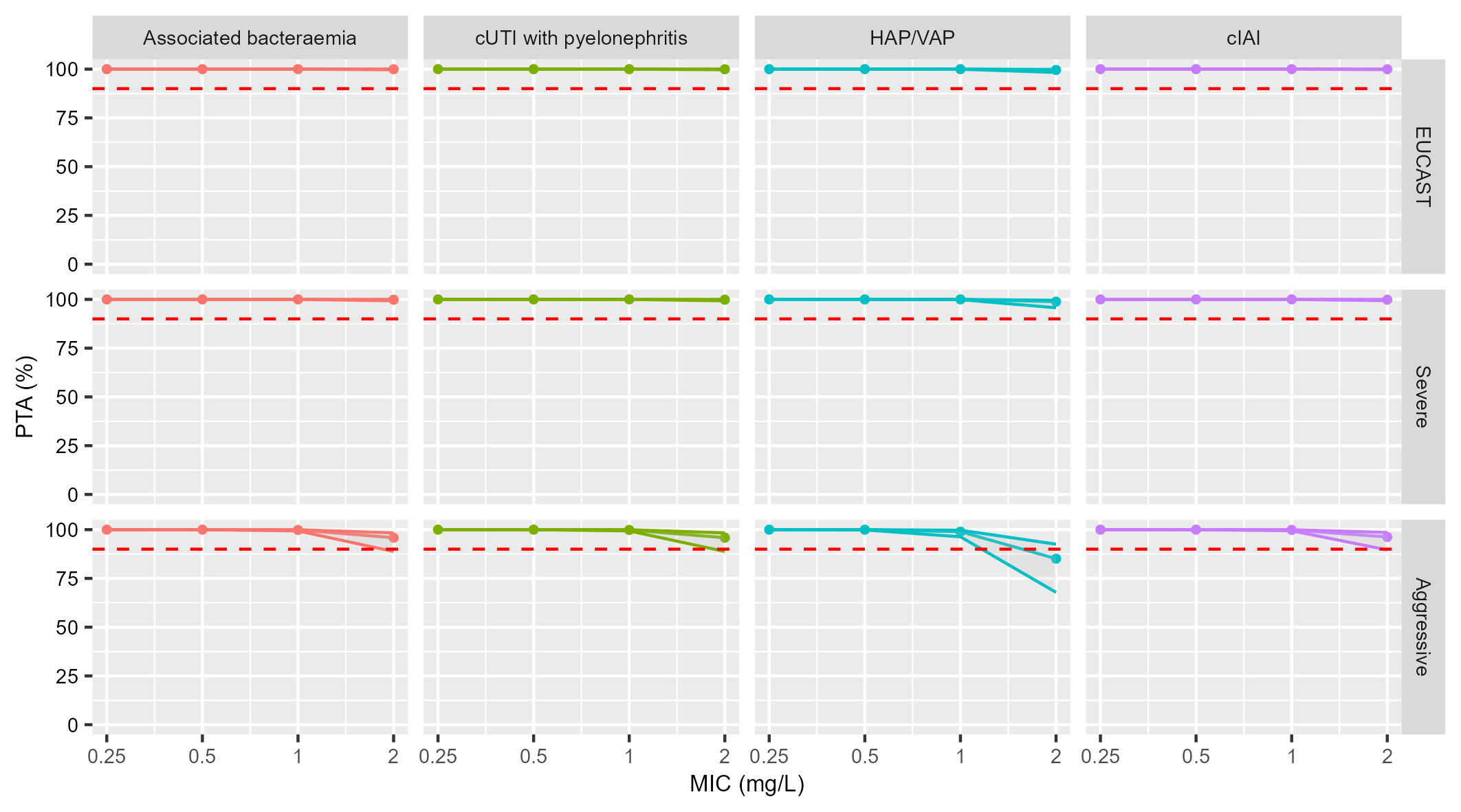
**

**Figures S4.** Probability of target attainment results for imipenem-relebactam (IMI-REL) in the TRC population, using high and low values of reported tissue penetration ratio and unbound fraction. Dotted lines correspond to reported values in the literature.

**
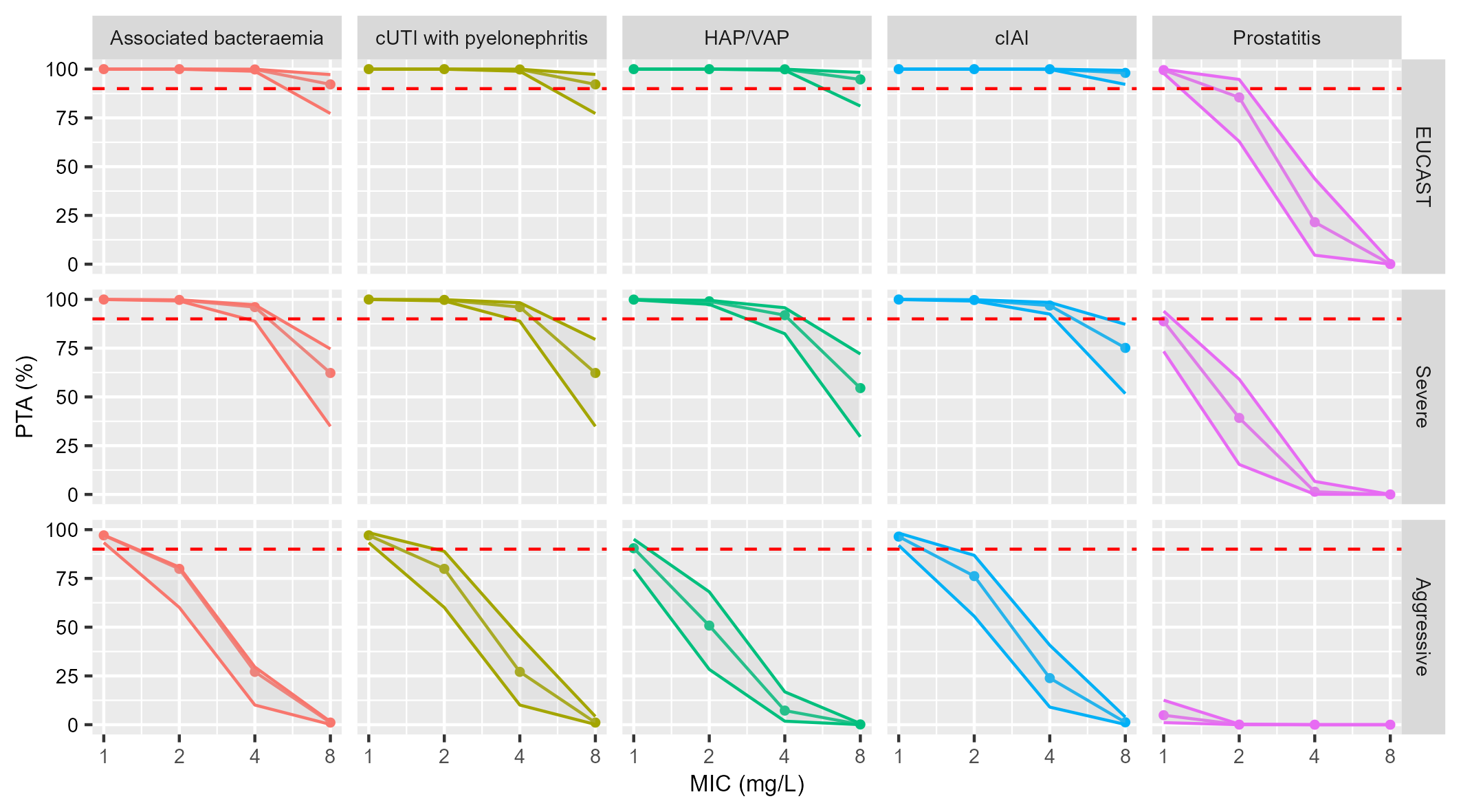
**

**Figures S5.** Probability of target attainment results for meropenem-vaborbactam (MER-VAB) in the TRC population, using high and low values of reported tissue penetration ratio and unbound fraction. Dotted lines correspond to reported values in the literature.

**
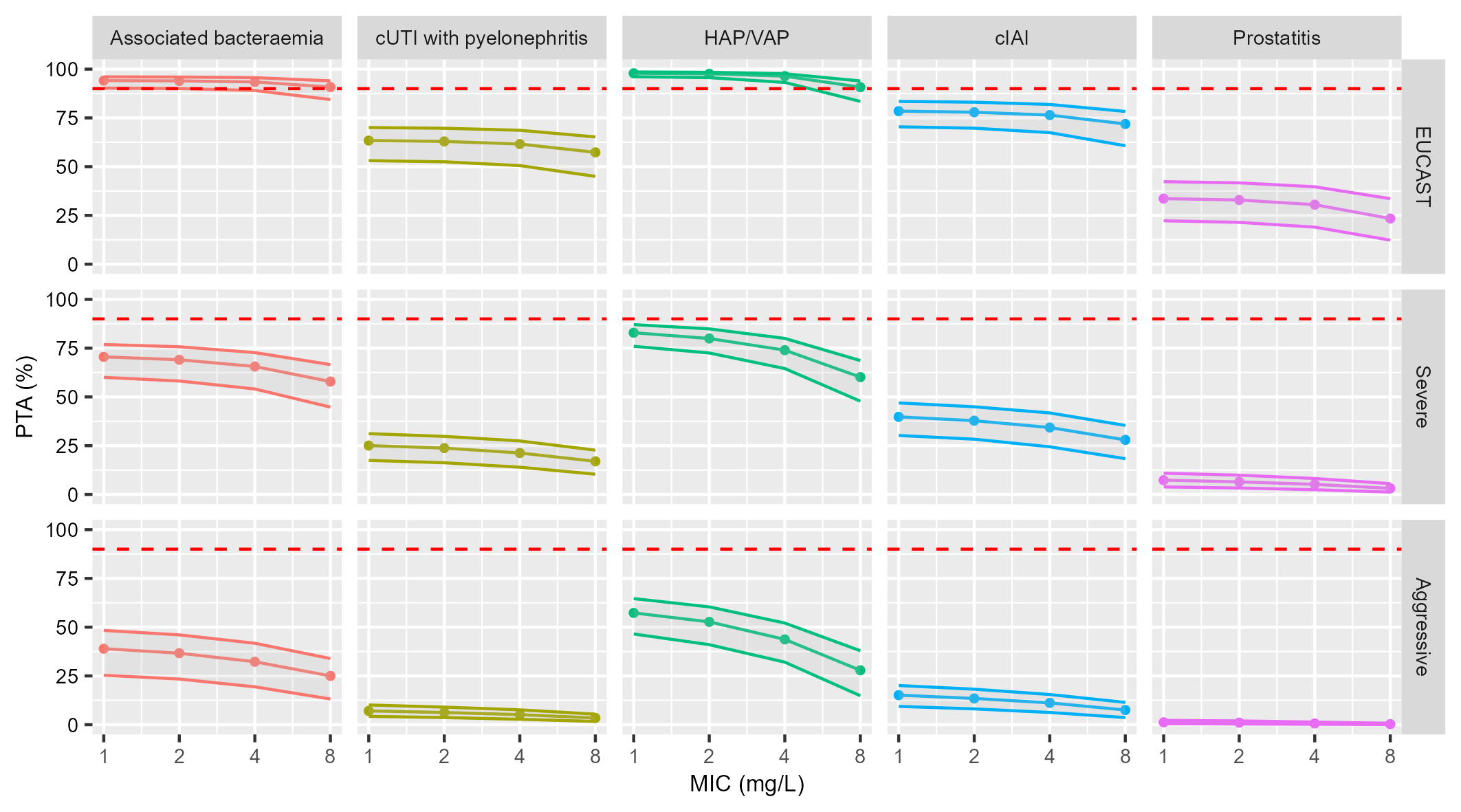
**

**Figures S6.** Probability of target attainment results for piperacillin-tazobactam (PIP-TAZ) in the TRC population, using high and low values of reported tissue penetration ratio and unbound fraction. Dotted lines correspond to reported values in the literature.

**References**

1. Li J, Lovern M, Green ML, *et al.* Ceftazidime‐Avibactam Population Pharmacokinetic Modeling and Pharmacodynamic Target Attainment Across Adult Indications and Patient Subgroups. *Clin Transl Sci* 2019; **12**: 151–63.

2. Zhang Z, Patel YT, Fiedler‐Kelly J, Feng H, Bruno CJ, Gao W. Population Pharmacokinetic Analysis for Plasma and Epithelial Lining Fluid Ceftolozane/Tazobactam Concentrations in Patients With Ventilated Nosocomial Pneumonia. *J Clin Pharmacol* 2021; **61**: 254–68.

3. Bhagunde P, Patel P, Lala M, *et al.* Population Pharmacokinetic Analysis for Imipenem-Relebactam in Healthy Volunteers and Patients With Bacterial Infections. *CPT Pharmacometrics Syst Pharmacol* 2019; **8**: 748–58.

4. Trang M, Griffith DC, Bhavnani SM, *et al.* Population Pharmacokinetics of Meropenem and Vaborbactam Based on Data from Noninfected Subjects and Infected Patients. *Antimicrobial Agents and Chemotherapy* **65**: e02606-20.

5. Udy AA, Lipman J, Jarrett P, *et al.* Are standard doses of piperacillin sufficient for critically ill patients with augmented creatinine clearance? *Crit Care* 2015; **19**. Available at: https://www.ncbi.nlm.nih.gov/pmc/articles/PMC4341874/. Accessed November 10, 2021.

6. Greppmair S, Brinkmann A, Roehr A, *et al.* Towards model-informed precision dosing of piperacillin: multicenter systematic external evaluation of pharmacokinetic models in critically ill adults with a focus on Bayesian forecasting. *Intensive Care Med* 2023; **49**: 966–76.

7. European Medicines Agency. Zavicefta (ceftazidime-avibactam): Summary of products characteristics. Available at: https://www.ema.europa.eu/en/documents/product-information/zavicefta-epar-product-information_en.pdf.

8. European Medicines Agency. Zerbaxa (ceftolozane-tazobactam): Summary of products characteristics. Available at: https://www.ema.europa.eu/en/documents/product-information/zerbaxa-epar-product-information_en.pdf.

9. European Medicines Agency. Recarbrio (imipenem-cilastatin-relebactam): Summary of products characteristics. Available at: https://www.ema.europa.eu/en/documents/product-information/recarbrio-epar-product-information_en.pdf.

10. European Medicines Agency. Vaborem (meropenem-vaborbactam): Summary of products characteristics. Available at: https://www.ema.europa.eu/en/documents/product-information/vaborem-epar-product-information_en.pdf.

11. European Medicines Agency. Tazocin (piperacillin-tazobactam): Summary of products characteristics. Available at: https://www.ema.europa.eu/en/documents/referral/tazocin-article-30-referral-annex-iii_en.pdf.

12. Sou T, Hansen J, Liepinsh E, *et al.* Model-Informed Drug Development for Antimicrobials: Translational PK and PK/PD Modeling to Predict an Efficacious Human Dose for Apramycin. *Clinical Pharmacology & Therapeutics* 2021; **109**: 1063–73.

13. European Committee on Antimicrobial Susceptibility Testing. Ceftazidime-avibactam: Rationale for the clinical breakpoints, version 1.0. 2020. Available at: http://www.eucast.org.

14. European Committee on Antimicrobial Susceptibility Testing. Ceftolozane/Tazobactam: Rationale for the clinical breakpoints, version 1.0. 2020. Available at: http://www.eucast.org.

15. European Committee on Antimicrobial Susceptibility Testing. Imipenem-relebactam Rationale Document, version 1.0. 2021. Available at: http://www.eucast.org.

16. European Committee on Antimicrobial Susceptibility Testing. Meropenem-Vaborbactam Rationale Document, version 1.0. 2021. Available at: http://www.eucast.org.

17. European Committee on Antimicrobial Susceptibility Testing. Piperacillin-tazobactam: Rationale for the clinical breakpoints, version 1.0. 2010. Available at: http://www.eucast.org.

18. Buijk SLCE, Gyssens IC, Mouton JW, Van Vliet A, Verbrugh HA, Bruining HA. Pharmacokinetics of ceftazidime in serum and peritoneal exudate during continuous versus intermittent administration to patients with severe intra-abdominal infections. *J Antimicrob Chemother* 2002; **49**: 121–8.

19. Abbas AM, Taylor MC, Da Silva C, Francis RA, Bennet C. Penetration of ceftazidime into the human prostate gland following intravenous injection. *J Antimicrob Chemother* 1985; **15**: 119–21.

20. Yoshimura K, Ohge H, Ikawa K, *et al.* Ceftolozane–Tazobactam Pharmacokinetics in the Abdominal Tissue of Patients Undergoing Lower Gastrointestinal Surgery: Dosing Considerations Based on Site-Specific Pharmacodynamic Target Attainment. *Infect Dis Ther* 2023; **12**: 193–207.

21. Ikawa K, Morikawa N, Sakamoto K, *et al.* Pharmacokinetics and Pharmacodynamic Assessment of Imipenem in the Intraperitoneal Fluid of Abdominal Surgery Patients. *CHE* 2008; **54**: 131–9.

22. Soga Y, Ohge H, Ikawa K, Morikawa N, Ikeda K, Sueda T. Peritoneal pharmacokinetics and pharmacodynamic target attainment of meropenem in patients undergoing abdominal surgery. *J Chemother* 2010; **22**: 98–102.

23. Nishikawa G, Ikawa K, Nakamura K, *et al.* Prostatic penetration of meropenem in humans, and dosage considerations for prostatitis based on a site-specific pharmacokinetic/pharmacodynamic evaluation. *Int J Antimicrob Agents* 2013; **41**: 267–71.

24. Murao N, Ohge H, Ikawa K, *et al.* Pharmacokinetics of piperacillin-tazobactam in plasma, peritoneal fluid and peritoneum of surgery patients, and dosing considerations based on site-specific pharmacodynamic target attainment. *Int J Antimicrob Agents* 2017; **50**: 393–8.

25. Kobayashi I, Ikawa K, Nakamura K, *et al.* Penetration of piperacillin-tazobactam into human prostate tissue and dosing considerations for prostatitis based on site-specific pharmacokinetics and pharmacodynamics. *J Infect Chemother* 2015; **21**: 575–80.
